# Editorial: Metabolic adjustments and gene expression reprogramming for symbiotic nitrogen fixation in legume nodules, volume II

**DOI:** 10.3389/fpls.2023.1141269

**Published:** 2023-01-24

**Authors:** Kejing Fan, Brett James Ferguson, Nacira Belen Muñoz, Man-Wah Li, Hon-Ming Lam

**Affiliations:** ^1^ Center for Soybean Research of The State Key Laboratory of Agrobiotechnology, School of Life Sciences, The Chinese University of Hong Kong, Shatin, Hong Kong SAR, China; ^2^ Integrative Legume Research Group, School of Agriculture and Food Sciences, The University of Queensland, St Lucia, Brisbane, QLD, Australia; ^3^ National Institute of Agricultural Technology, Córdoba, Argentina

**Keywords:** legume, nitrogen fixation, nod factor hydrolase, non-coding RNA (ncRNA), nodulation, proteome, transcription regulation, translatome

The increased demand for food and more sustainable agricultural practices have brought increased attention to legume crops, a family of plants that are able to carry out symbiotic nitrogen fixation which provides a reliable source of bioavailable nitrogen. Thus, the cultivation of legumes can effectively reduce the reliance on the not-so-environmentally friendly synthetic nitrogen fertilizer. The legume-rhizobium symbiosis results in the formation of new root organs called nodules, where rhizobia differentiate into bacteroids and carry out symbiotic nitrogen fixation to reduce the atmospheric nitrogen (N_2_) to ammonia, which can then be used by the host plant (Ferguson et al., 2010; Liu et al., 2018; Roy et al., 2020; Yang et al., 2022). Much effort has been dedicated to researching the legume-rhizobium symbiosis and nitrogen fixation mechanism to understand and improve these beneficial processes.

Nodulation and nitrogen fixation are tightly regulated to balance between the benefits of nitrogen and other critical resources (Ferguson et al., 2019). Nodulation is initiated by the exchange of signals between the plant and the rhizobium (Ferguson and Mathesius, 2014; Yang et al., 2022). Typically, the legume host secretes flavonoids or their derivatives into the rhizosphere to attract the rhizobium (Sprent et al., 2017). The flavonoids induce the rhizobium to release nod factors (NFs), a group of lipochiooliosaccharides, which trigger the transcriptomic reprogramming of the host to initiate the nodulation process (Yang et al., 2022). Nevertheless, the host can choose either to accept the NFs through NF receptors or to decline them by hydrolyzing the NFs into non-intimidating forms (Staehelin et al., 1994; Goormachtig et al., 1998; Ovtsyna et al., 2007; Tian et al., 2013; Zhang et al., 2016). A class-V chitinase (MtCHIT5b) was recently characterized in *Medicago truncatula*, which can cleave the NF from *Sinorhizobium meliloti* in the rhizosphere (Li et al.). Increasing MtCHIT5b in the rhizosphere through overexpression led to a significant reduction in nodule formation, demonstrating the NF hydrolase to be a key player at the frontier of the interaction between the legume and the rhizobium for nodulation.

When NFs are successfully perceived by NF receptors, the host cells will undergo massive transcriptomic reprogramming governed by a cocktail of transcription factors for the subsequent organogenesis and nitrogen fixation. The respective subunits of heterotrimeric Nuclear Factor Y (NF-Y) transcription factors have been demonstrated to play crucial roles in nodule formation (Wang et al., 2015; Shrestha et al., 2021). Such an interaction, as that between NF-Y subunit C1 (NF-YC1) and the NF-YC1-interacting protein kinase (NIPK) from *Phaseolus vulgaris*, was recently identified through yeast two-hybrid screening (Clúa et al.). Although NIPK does not possess the canonical residues for the catalytic activities of kinases, its localization to the plasma membrane and the cytoplasm suggests that it may regulate the function of NF-YC1 through the re-localization of the transcription factor protein. The knockdown of *NIPK* had significant negative impacts on nodule number and nodule size, resembling the phenotype of *NF-YC1*-RNAi plants. The silencing of *NIPK* also impaired the induction of cell cycle-related and nodulation-related genes responsive to rhizobium inoculation, suggesting NIPK’s involvement in the transcription regulation of legume-rhizobium symbiosis.

The translation of the open reading frames of transcripts collectively determines the proteome. Song et al. utilized translating ribosome affinity purification (TRAP) sequencing to capture the actively translated transcripts in the root cortex of soybean in response to *Bradyrhizobium japonicum* inoculation. They have not only found the well-known nodulation marker genes, but have also discovered a new set of genes potentially important for early nodulation events. These discoveries enable legume researchers to further resolve the interconnected regulation networks for nodule formation.

As an essential macronutrient, phosphorus (P) influences various vital functions in plants. Deficiency in P can be detrimental to nodulation and nitrogen fixation. The effects of different P regimes on the proteome of soybean nodules were determined using a high-throughput proteomic approach (Yao et al.). While comparing nodules with normal P to those with P deficiency, the most affected gene ontologies (GO) and pathways of the differentially expressed proteins (DEP) are mainly related to protein translation. At the other end, carbon and nitrogen metabolism-related GO and pathways are the most affected under high-P conditions. These findings enhance our understanding of how nodules respond to different P conditions at the proteomic level.

Recent evidence revealed that non-coding RNAs (ncRNAs), including microRNAs (miRNAs), long non-coding RNAs (lncRNAs) and circular RNAs (circRNAs), form another layer of regulation of nodulation and nitrogen fixation by controlling the transcription and translation of effector genes (Yan et al., 2013; Yan et al., 2015; Fan et al., 2021). Fan et al. summarized and discussed the current understanding of ncRNAs in legume nodulation and nitrogen fixation in root nodules, focusing on the regulation of hormone signal transduction, the autoregulation of nodulation (AON) pathway and nutrient homeostasis in nodules by ncRNAs. They also touched on the potential applications of ncRNAs in agriculture. Unraveling the roles of ncRNAs in legume nodulation will provide new insights into designing better leguminous crops for sustainable agriculture.

This Research Topic explores the new findings in NF signal transduction, nodule development, nodule metabolic changes upon P stress, and technological breakthroughs in translatomic analyses in nodule formation. The contributing articles collectively shed light on the molecular mechanisms involved in these areas and provide new research directions and scenarios for rhizobium-legume symbiosis.

## Author contributions

H-ML coordinated the preparation and completed the final version of this editorial. KF and M-WL wrote the first draft. BF and NM contributed to the editing of this Research Topic and proofreading this editorial. All authors contributed to the article and approved the submitted version.
